# Nanostructured photoelectrochemical solar cell for nitrogen reduction using plasmon-enhanced black silicon

**DOI:** 10.1038/ncomms11335

**Published:** 2016-04-20

**Authors:** Muataz Ali, Fengling Zhou, Kun Chen, Christopher Kotzur, Changlong Xiao, Laure Bourgeois, Xinyi Zhang, Douglas R. MacFarlane

**Affiliations:** 1 School of Chemistry, Monash University, Clayton, Victoria 3800, Australia; 2 Monash Centre for Electron Microscopy, Department of Materials Engineering, Monash University, Clayton, Victoria 3800, Australia

## Abstract

Ammonia (NH_3_) is one of the most widely produced chemicals worldwide. It has application in the production of many important chemicals, particularly fertilizers. It is also, potentially, an important energy storage intermediate and clean energy carrier. Ammonia production, however, mostly uses fossil fuels and currently accounts for more than 1.6% of global CO_2_ emissions (0.57  Gt in 2015). Here we describe a solar-driven nanostructured photoelectrochemical cell based on plasmon-enhanced black silicon for the conversion of atmospheric N_2_ to ammonia producing yields of 13.3 mg m^−2^ h^−1^ under 2 suns illumination. The yield increases with pressure; the highest observed in this work was 60 mg m^−2^ h^−1^ at 7 atm. In the presence of sulfite as a reactant, the process also offers a direct solar energy route to ammonium sulfate, a fertilizer of economic importance. Although the yields are currently not sufficient for practical application, there is much scope for improvement in the active materials in this cell.

## Abstract (web summary)

In nature, nitrogen fixation is achieved via light-dependent nitrogenases, but industrial photochemical conversion of nitrogen into ammonia has so far proven inefficient. Here, the authors describe a nanostructured black silicon photoelectrochemical cell that can catalyse the process using solar energy.

## Graphical abstract

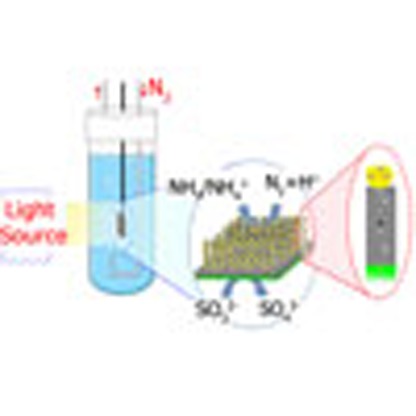

Ammonia production is a highly energy intensive process, consuming 1–3% of the world electrical energy and ∼5% of the world natural gas production^1^,^2^. World production is around 200 million tonnes annually^2^,^3^, reflecting the vast need for this chemical in agriculture, pharmaceutical production and many other industrial processes. Ammonia is also being considered as a carbon-free solar energy storage material, due to its useful characteristics as a chemical energy carrier. Compared with other chemicals that could be used to store solar energy (such as hydrogen), ammonia is safe, ecofriendly and, most importantly, produces no CO_2_ emissions^4^. Apart from the well-known industrial Haber–Bosch process, there are a number of other chemical, electrochemical and biological production methods^5^,^6^,^7^,^8^,^9^. Among them, an electrochemical method is of considerable interest because it can be coupled with hydro, wind, solar or nuclear energy. The electrochemical reduction of nitrogen largely depends on the structure, components and surface morphology of the electrocatalyst^10^. Polyaniline electrodes have been investigated in methanol/LiClO_4_/H^+^ solution, achieving a maximum current efficiency of 16% at −0.12 V (versus standard hydrogen electrode (NHE)) at room temperature and elevated pressure^11^. By using a membrane electrode assembly based cell with Pt electrodes, an ammonia production rate of 1.14 × 10^−5^mol m^−2^ s^−1^ has been achieved from air and water at ambient temperature and pressure at an overall cell voltage of 1.6 V (ref. 12). By using the mixture of air and steam in a molten hydroxide suspension of nano-Fe_2_O_3_, ammonia has also been produced at a cell voltage of 1.2 V at coulombic efficiency of 35% (ref. 13). Although this advance shows a great promise in competing with current ammonia industry, the high temperature (∼200 °C) used still requires extra input of heat and energy. Thus, there is intense interest in new catalysts for nitrogen reduction processes and alternative means of carrying out the reaction^14^,^15^,^16^.

Photochemical conversion provides a promising approach to convert nitrogen into ammonia by using solar energy. Early attempts used titania, or modified titania semiconductor catalysts^17^,^18^,^19^, but all of these produced only impractically low efficiencies. The recent development of surface plasmon resonance (SPR) has provided some new opportunities. Plasmon-induced ammonia synthesis has been demonstrated through nitrogen photofixation on a gold nanoparticle (GNP) -coated Nb-SrTiO_3_ substrate with visible light irradiation^20^. Photo-illuminated diamond has also been used as a solid-state source of solvated electrons in water for nitrogen reduction^21^. Photochemical nitrogen conversion to ammonia has been achieved on chalcogels containing FeMoS inorganic clusters in ambient conditions; the significance of this work lies in that Fe and Mo are two key metals found in nitrogenases that catalyse nitrogen reduction in nature^22^. More recently, oxygen vacancies of BiOBr nanosheets have been investigated for nitrogen reduction; this work in particular offers improved yields, but suffers from a decreasing photo-reactivity due to the quench of oxygen vacancies^23^.

Black silicon (bSi) is a form of silicon in which its surface is covered by a layer of nanostructures (usually nanowires, nanorods or nanotips), which effectively suppresses reflection, by enhancing the scattering and absorption of light. As a consequence, the silicon wafers appear black, instead of the silver-grey typical of planar silicon wafers. bSi possesses many attractive properties, including low reflectance, a large and chemically active surface area, super-hydrophobicity, and a high luminescence efficiency when surface-feature sizes are reduced to a few nanometers^24^,^25^,^26^. In this work we have achieved solar light driven conversion of nitrogen to ammonia using a photoelectrochemical structure, based on plasmon-enhanced bSi, as the photo absorber, decorated with GNPs as the reduction catalysis sites and a hole-sink layer of Cr. This multi-layer structure creates an autonomous electrochemical device capable of carrying out oxidation and reduction reactions on different areas of the device, powered by photo-excitation.

## Results

### Fabrication of the photoelectrochemical cell

**Figure 1:**
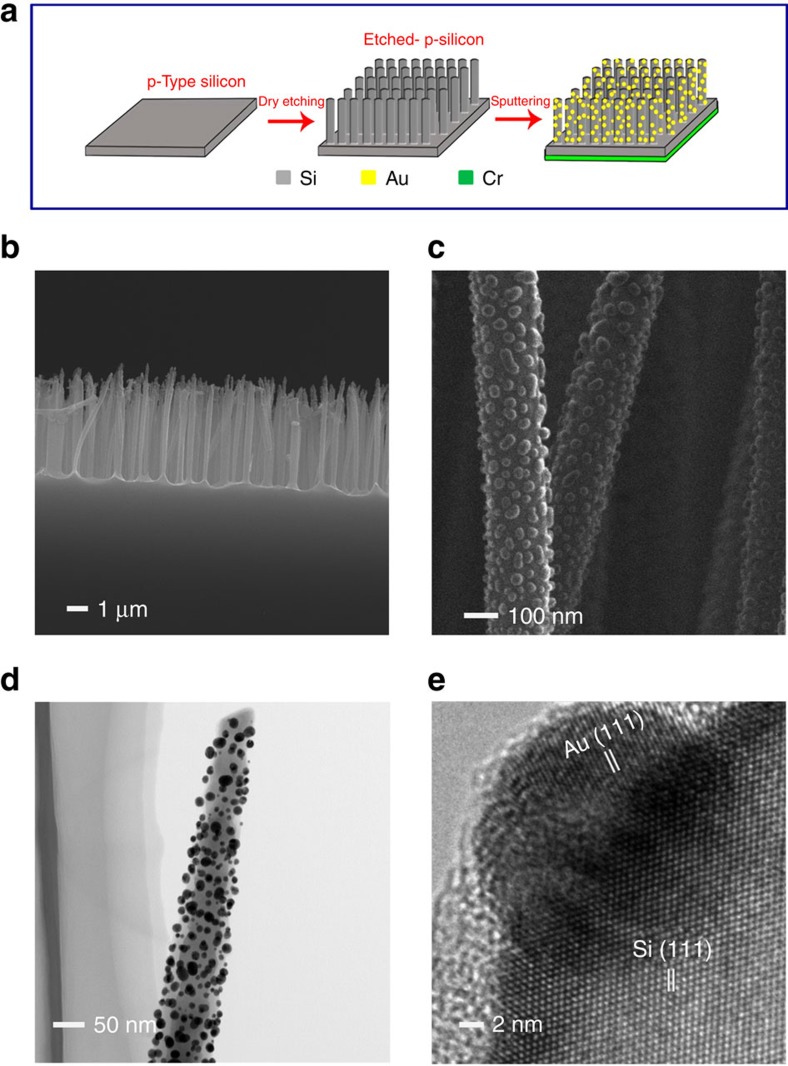
Schematic of fabrication and SEM and TEM images of the electrodes. (**a**) Schematic illustration of fabrication of the cell. (**b**) Cross-sectional view and (**c**) magnified view of SEM images of GNPs-coated black silicon nanostructure. (**d**) Corresponding TEM and (**e**) HRTEM images of GNPs-coated silicon nanowire. The well-resolved lattice spacings of 0.31 and 0.23 nm correspond to the Si {111} and Au {111} atomic planes, respectively. SEM, scanning electron micrograph; HRTEM, high-resolution TEM; TEM, transmission electron micrograph.

A schematic illustration of the fabrication steps and working mechanism of the photoelectrochemical cell and corresponding scanning electron micrograph and transmission electron micrographs are shown in Fig. 1 and Supplementary Figs 1 and 2. A p-type boron-doped commercial <100> silicon wafer was used as a substrate material. bSi was fabricated using a dry etching method. A GNP layer was then sputtered onto the etched surface as the photocathode. Finally, a Cr layer with thickness ∼50 nm was sputtered onto the back surface of the silicon wafer as an anode. Full experimental detail is provided in Methods section.

### Photoelectrochemical reduction of nitrogen

**Figure 2:**
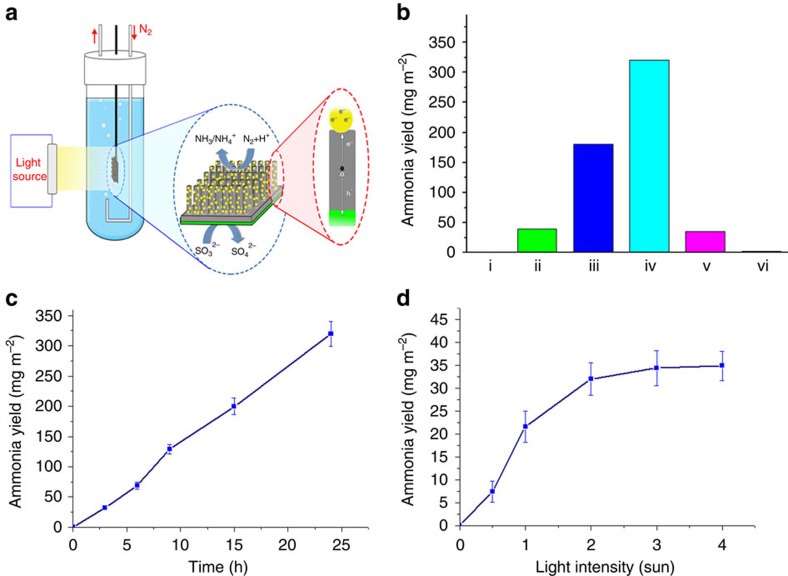
Photoelectrochemical nitrogen reduction. (**a**) Schematic diagram of the cell. (**b**) Yield of ammonia over 24 h obtained on different substrates: (i) P-type silicon, (ii) bSi, (iii) GNP/bSi, (iv) GNP/bSi/Cr and (v) Au/Si/Cr after illumination with two suns and (vi) GNP/bSi/Cr in dark. (**c**) The time-dependence of ammonia yield obtained after illumination with two suns (error bars are the s.d. of at least three replicates of independent measurement). (**d**) The light intensity-dependence of ammonia yield obtained after illumination for 3 h (error bars are an estimate of the combined errors of measurements).

A nitrogen photo-reduction cell was constructed (Fig. 2a) with nitrogen gas bubbling over the surface of the material and artificial solar light (300 W Xe lamp) as an illumination source. The yield of ammonia was measured by using an ammonia/ammonium ISE and the indophenol method^27^.

Control experiments were conducted in H_2_O (18.2 MΩ cm). Yields obtained over a 24 h period are shown in Fig. 2b. Ammonia production can be observed at a low level on bSi; however, after coating with GNPs, the yield of ammonia is increased by nearly four times. To avoid silicon oxidation and enhance charge separation, a Cr layer was coated on silicon to act as the hole-sink and anode; in other words, Cr facilitates hole collection from the Si and acts in this case as a sacrificial anode. This GNP/bSi/Cr photoelectrochemical cell exhibits yield of 320 mg m^−2^ over 24 h. However, the oxidation of Cr was also observed, confirming its role as a sacrificial hole-sink in the process. To avoid the loss of Cr, sodium sulfite was added to the electrolyte used to provide an alternate electron donor to scavenge the photogenerated holes and hence protect the Cr anode. Ammonia production as a function of time with this GNP/bSi/Cr/sulfite structure is shown in Fig. 2c; the final yield of ammonia under these conditions over a 24 h period was 320 mg m^−2^ or 13.3 mg m^−2^ h^−1^. A durability test consisting of repeated 3 h runs using this sodium sulfite electrolyte also showed very reproducible and stable behaviour for up to 18 h (Supplementary Fig. 3). These results show that the GNP/bSi/Cr cell exhibits excellent activity and stability for ammonia production compared with previous reports, as summarized in Supplementary Table 1. A control experiment was also performed to confirm that the source of the nitrogen in the ammonia was the bubbled N_2_, by using Ar to replace N_2_ in the experiment; no ammonia was detected (Supplementary Fig. 4).

While a number of possible sacrificial reagents could be used in this context, sulfite was chosen on the basis that, when used in its aqueous acid form, the product of the overall reaction can be ammonium sulfate: N_2_+3H_2_SO_3_+3H_2_O→2H_2_SO_4_+(NH_4_)_2_SO_4._ Equivalent reactions can use potassium or ammonium bisulfite in this process (as described in Supplementary Note 1). The final 24 h yield in terms of ammonium sulfate as a product corresponds to 1.24 g m^−2^. Since the production rate appears to be quite constant over the 24 h period of the experiments in Fig. 2c, it would appear that this yield is not limited by product buildup under these conditions (Supplementary Fig. 5). The effect of light intensity is shown in Fig. 2d, the ammonia yield increases linearly with the intensity of light up to 300 mW cm^−2^ and then plateaus. This limiting rate is likely to be related to factors such as the rates of mass transport of the reactants and products at the active surfaces.

**Figure 3:**
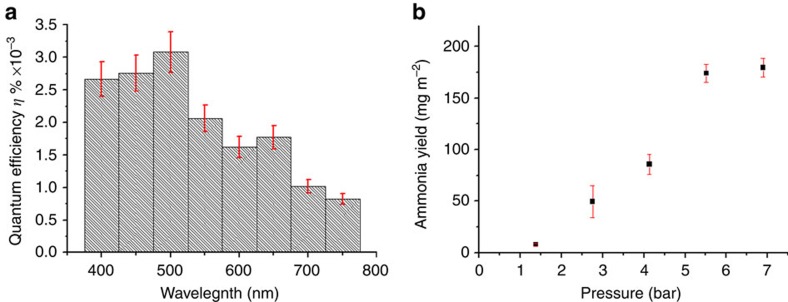
Quantum efficiency and high-pressure yield obtained on a GNP/bSi/Cr photoelectrochemical cell. (**a**) Quantum efficiency of ammonia synthesis on a GNP/bSi/Cr photoelectrochemical cell as a function of wavelength (error bars are estimates of the combined errors of measurements). (**b**) Yield of ammonia in three hours as a function of nitrogen gas pressure at two suns illumination in a fixed volume glass reactor (error bars are the s.d. of at least three replicates of independent measurement).

Quantum yield experiments (Fig. 3a) were carried out with 50-nm-wide bandpass filters, confirming that the nitrogen reduction can occur in whole visible range, dropping away as the band gap energy of Si is approached. A small absorption maximum that is observed in the region of the SPR region for GNPs (Supplementary Fig. 6) also appears in the region of 500 nm in Fig. 3a. This suggests that excitation of surface plasmons in the GNP particles provides an additional photo-excitation mechanism that contributes to the overall yield in this region of the spectrum, as has been observed in other SPR-enhanced processes^28^,^29^. To understand the relationship between the dissolved amounts of nitrogen in the solution and the generated ammonia-ammonium yield, the effect of N_2_ pressure on the reaction was investigated in a closed glass pressure vessel (Fig. 3b). An approximately linear dependence of yield on pressure was observed over the range studied, and the yield was 60 mg m^−2^ h^−1^ at 7 atm, as would be expected from a Henry's law dependence of nitrogen solubility on pressure in the aqueous medium. This suggests that the concentration of nitrogen at the reactive sites on the surface is a rate limiting factor in these experiemnts. On the other hand, in our ambient condition experiments, bubbling nitrogen gas generates strong agitation of the medium created near the surface as well as a dynamic gas–liquid–solid interface, which enhances reactions rates by facilitating transport to and from the reaction sites in the strucure (this also explains the relatively lower yields at lower pressures in Fig. 3b). This suggests that strategies to further enhance yield in either case could seek to enhance the kinetics of these processes via further manipulation of the nanostructure of the bSi.

## Discussion

The various reference experiments in Fig. 2b reveal the roles of each layer in the overall reaction process, as illustrated in Supplementary Fig. 7. When the bSi was replaced by unetched pristine silicon, the ammonia yield was only ∼11% of that produced by the GNP/bSi/Cr structure. bSi effectively suppresses reflection, while simultaneously enhancing the scattering and absorption of light. bSi also provides an extremely large surface area for decoration by the GNPs. High activities of silicon nanostructures have been demonstrated previously in both photoelectrolytic and chemical water splitting^30^,^31^. The GNP-coated bSi material shows much superior ammonia yield over bSi alone, revealing the functions of the GNPs in separating charge and as an electrocatalytic site for the N_2_ reduction reaction. Meanwhile, bSi facilitates hole transfer to the anode layer (Cr) to execute the oxidation reactions. After coating with Cr on the backside, the ammonia production of the GNP/bSi/Cr cell increases to about two times that of GNP/bSi and eight times that of pure bSi. The detailed reduction mechanism of N_2_ to NH_3_, which involves six electrons and six protons, is not yet well-understood and is the focus of ongoing experimental and theoretical investigations by a number of groups^6^,^10^,^32^, including ourselves. It is important to note that the overall reaction in the dark with sulfite is not spontaneous, as indicated by the free energy calculation shown in the Supplementary Note 2. This confirms that role of the cell is energy injection into the process, as opposed to photo-catalytic. It is worth noting that sulfite is a product of coal-fired power station flue gas scrubbing, while ammonium sulfate is a commonly used form of ammonia as a fertilizer. So this autonomous photoelectrochemical process may offer scope to valorize this otherwise hazardous waste product.

In summary, we describe a nanostructured photoelectrochemical cell that is capable of mimicking the nitrogen fixation and conversion process of nitrogenases in nature and producing ammonia (13.3 mg m^−2^ h^−1^, at 2 suns) and an ammonia based fertilizer in a fully solar-driven process. The photoelectrochemical cell is not inherently area limited and can be scaled up with the silicon wafer size, and the nanostructures of the cell can be further improved and optimized in term of the component, size and configuration. Hence significant potential exists for further development of this approach to ammonia generation by using standard manufacturing process. At this preliminary stage, the efficiency of our photoelectrochcmical process is still too low for practical ammonia manufacture. One of the possibilities to improve the efficiency is to couple our cell with a separate PV cell (as detailed further in Supplementary Table 2), since the configuration of our cell is ready to be utilized as an electrochemical cell. Finally, the combination of black silicon-based photo-adsorber and plasmonic catalysts described here could provide a pathway toward an unassisted solar-to-chemical conversion in many applications such as CO_2_ fixation and water splitting.

## Methods

### Preparation and characterization of black silicon (bSi) and electrodes

A p-type boron-doped 100 mm commercial <100> silicon wafer (resistivity=10–20 ohm cm) of 525 μm thickness (Atecom Ltd, Taiwan) was used as a substrate material for black silicon formation. The wafers were used as supplied. The dry etching process was carried out using an Oxford PlasmaLab 100 ICP380 system. The etching process was a mixed mode, wherein etching and passivation occurred at the same time. Process conditions for the black silicon formation were: SF_6_ gas flow rate 65 s.c.c.m., O_2_ gas flow rate 44 sccm, process pressure of 35 mTorr, 100 W reactive ion etching (RIE) power, 20 °C electrode temperature and 10 Torr He backside cooling pressure. The RIE process resulted in a homogeneously distributed silicon nanowire arrays with length about 3 μm across the full wafer after 20 min etching. Nitrogen adsorption–desorption experiments were performed at 77 K with a Micromeritics Tristar II. The samples were degassed at 200 C for 5 h before measurement. The surface area of bSi calculated from nitrogen sorption results by Brunauer–Emmett–Teller method is 10.4 cm^2^. After the RIE process, the gold coating was carried out on a K550X sputter coater at 25 mA at 1 × 10^−1^ mbar. The discharge time was 4 min. The size of the GNPs ranges from 3 to 30 nm. The prepared bSi with GNP on its surface was back coated with chromium; the coating process was carried out using a high-resolution turbomolecular-pumped sputter coater, including a TK8845 54 mm Ø × 0.3 mm chromium (Cr) target (Q150T S Quorum Technologies). The coating conditions were 40 mA current density for 120 s (2 × 60 s). The morphology and structure of bSi were investigated by scanning electron microscopy (scanning electron micrograph, JEOL JSM-7100) and transmission electron microscopy (transmission electron micrograph, JEOL-2100F).

### Photoelectrochemical nitrogen reduction

All of the chemical reagents in this study were of analytical grade and were supplied by Sigma-Aldrich (Australia).

A photo-reduction cell to reduce nitrogen into ammonia was constructed; the cell contained 10 ml deionized water or electrolyte as specified below and the nitrogen flow rate was 10 ml min^−1^. Nitrogen was passed through the cell for 1 h to remove oxygen. Black silicon pieces with size of 10 × 10 mm^2^ were tested. The artificial solar light source was a 300 W Xe lamp, while the illumination intensity used was 2 suns unless otherwise specified. The experimental conditions were as follows: *bSi (dark)*: black silicon, (reaction conditions; 10 ml deionized water, in dark). *Si:* pristine unetched silicon (reaction conditions; 10 ml deionized water, 2 suns). *GNP/bSi*: GNPs-coated black silicon, (reaction conditions; 10 ml deionized water, 2 suns). *GNP/bSi/Cr*: GNPs-coated black silicon (front) and chromium (back) (reaction conditions; 10 ml deionized water, 2 suns). *bSi/GNP/Cr+SO3−2*: GNPs-coated black silicon (front) and chromium (back), (reaction conditions; in 10 ml 150 p.p.m. sodium sulfite electrolyte, 2 suns). *GNP/Si/Cr*: GNPs-coated pristine unetched silicon (front) and chromium (back) (reaction conditions; 10 ml deionized water, 2 suns). *GNP/bSi/Cr(dark)*: GNPs-coated black silicon (front) and chromium (back) (reaction conditions; 10 ml deionized water, in dark). The cell solution was analysed directly for its ammonia content. The exiting nitrogen gas stream was bubbled through a water filled collector vessel to trap any ammonia carried by the nitrogen for analysis; the fraction appearing in the collector vessel was typically ∼12% and was included in the overall NH_3_ yield results of GNP/bSi/Cr cell in sodium sulfite electrolyte.

### High-pressure experiments

The p-GNP/bSi/Cr was tested under high-pressure nitrogen gas using a Q-tube purging-35-SS vessel, purchased from Q Labtech. The reaction was carried out under irradiation using a 300 W Xe lamp with the intensity equivalent to 2 suns. The electrolyte used was 10 ml deionized water and the catalyst size 1 cm^2^ (0.12 g). The electrolyte was bubbled with nitrogen for 20 min followed by addition to the reaction vessel which was purged with nitrogen gas before irradiation. Following irradiation for 3 h the concentration of ammonia/ammonium was analysed.

### Ammonia analysis

Ammonia products were analysed by using (i) an ammonia/ammonium ISE (YSI, 6883) and (ii) the indophenol method^33^. The YSI probe was calibrated using standard ammonia solutions containing the sacrificial agent (at concentration lower than electrode manufacturer's salinity limit). To ensure that the added SO_3_^2−^ did not interfere with the ammonia analysis methods in this work, the ISE was recalibrated using standard solutions containing the same amount of sacrificial agent (Supplementary Fig. 8). The indophenol method was used as a confirmation of the final yield of each experiment (supplementary Fig. 9). The UV–vis absorption peak of indophenol appears at ∼630 nm. At higher concentrations near the UV–vis spectrophotometer absorption limit, samples were diluted to ¼ or ½ of original concentration^34^,^35^.

## Additional information

**How to cite this article:** Ali, M. *et al*. Nanostructured photoelectrochemical solar cell for nitrogen reduction using plasmon-enhanced black silicon. *Nat. Commun.* 7:11335 doi: 10.1038/ncomms11335 (2016).

## Supplementary Material

Supplementary Information Supplementary Figures 1-9, Supplementary Tables 1-2, Supplementary Notes 1-2 and Supplementary References.
