# Supplementary material for: Nanostructured photoelectrochemical solar cell for nitrogen reduction using plasmon-enhanced black silicon
Source: Nat Commun. 2016 Apr 20;7:11335. doi: 10.1038/ncomms11335 (PMC4842983; doi:10.1038/ncomms11335)
Supplement: Supplementary Information — Supplementary Figures 1-9, Supplementary Tables 1-2, Supplementary Notes 1-2 and Supplementary References. [file ncomms11335-s1.pdf]

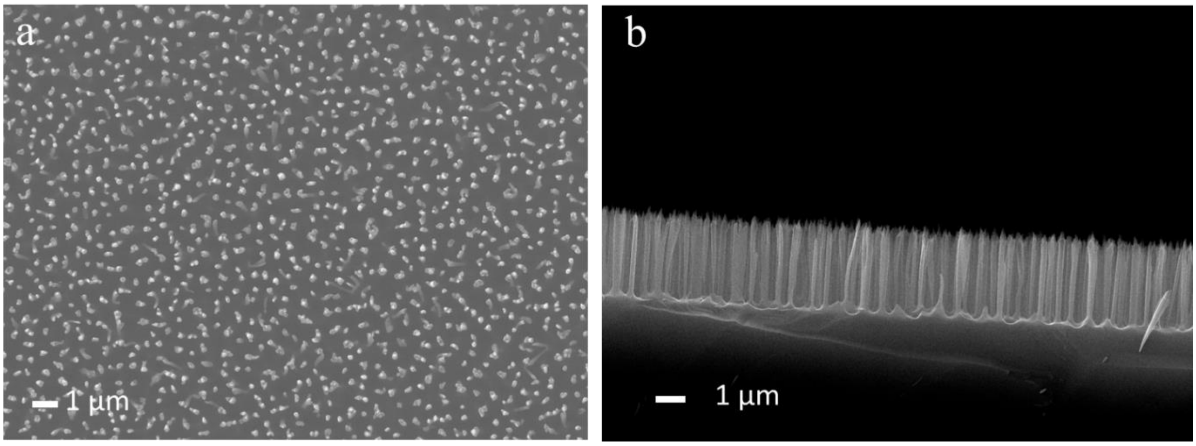

Supplementary Figure 1. SEM images of the bSi. (a) Plain view and (b) cross-sectional view.

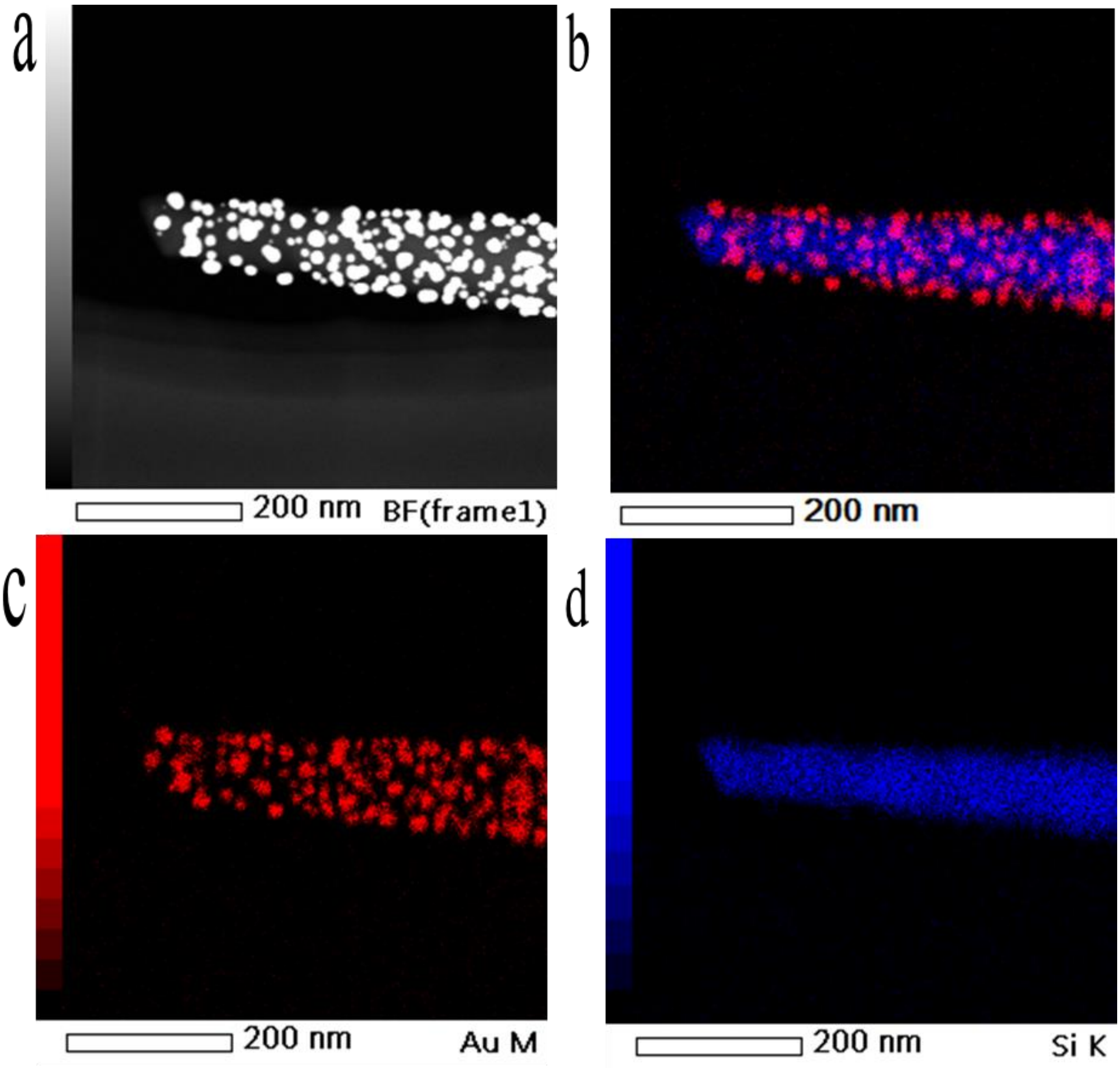

Supplementary Figure 2. (a) STEM and (b-d) EDX images of gold nanoparticles coated a silicon nanowire of the bSi.

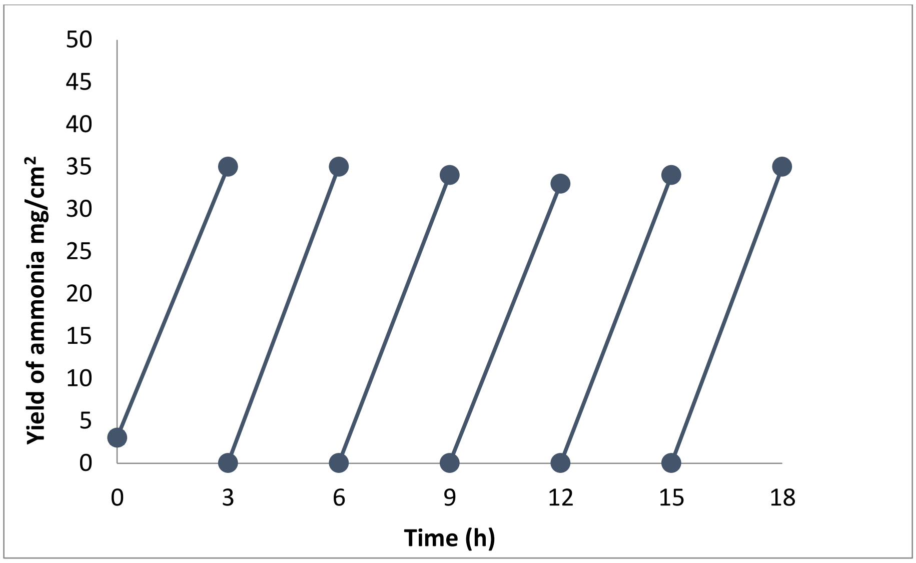

Supplementary Figure 3. Durability tests for the prepared catalysts. Blue points are for bSi/GNP/Cr sample with sulfite sacrificial agent. To ensure that depletion of the reactants or build-up of products did not affect the results, the solution has been replaced after each 3 hour period; the catalyst in this case remains stable over 6 cycles due to the action of the sacrificial agent in solution.

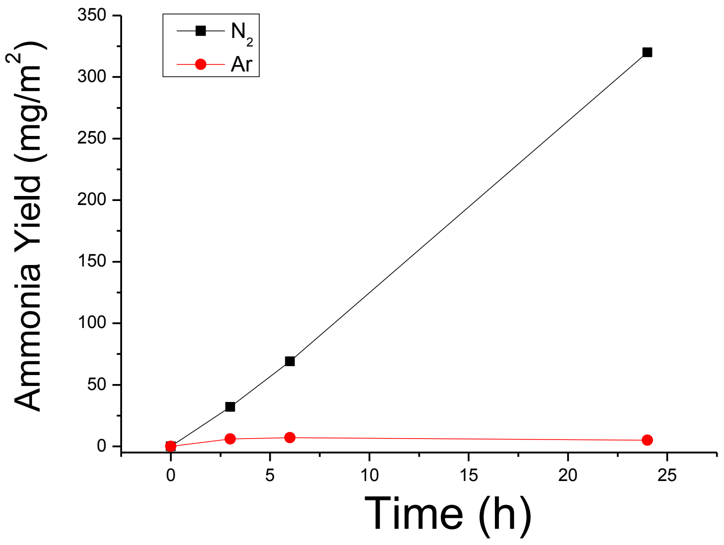

Supplementary Figure 4. Comparison of ammonia yields on GNP/bSi/Cr bubbled with (a) N_2 at 10 ml/min and (b) Ar at 10 ml/min. This control experiment confirms that the ammonia product is produced from the reduction of bubbled N_2.

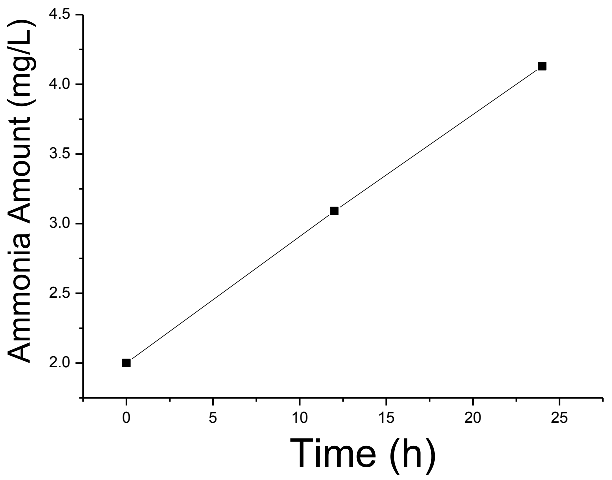

Supplementary Figure 5. The time-dependence of ammonia concentration obtained on GNP/bSi/Cr after illumination with 2 suns in solution containing 2 mg/L ammonia. This control experiment confirms that the photoelectrochemical cell can produce ammonia at constant rate even the ammonia concentration in the solution reaches certain level.

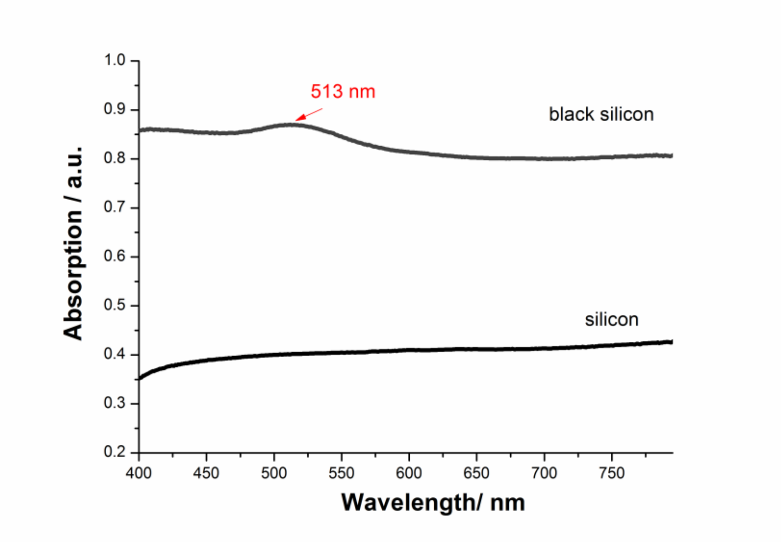

Supplementary Figure 6. UV-vis spectra of gold nanoparticle coated bSi compared with unetched Si. The difference in optical absorption is a result of the nano-structure modification created by the RIE.

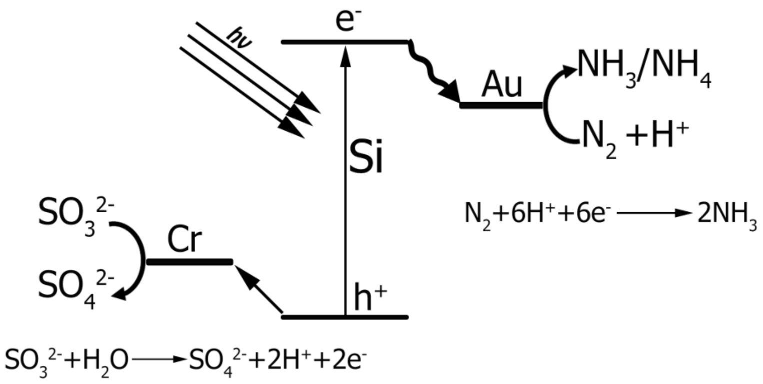

Supplementary Figure 7. Illustration of the electrochemical reaction processes.

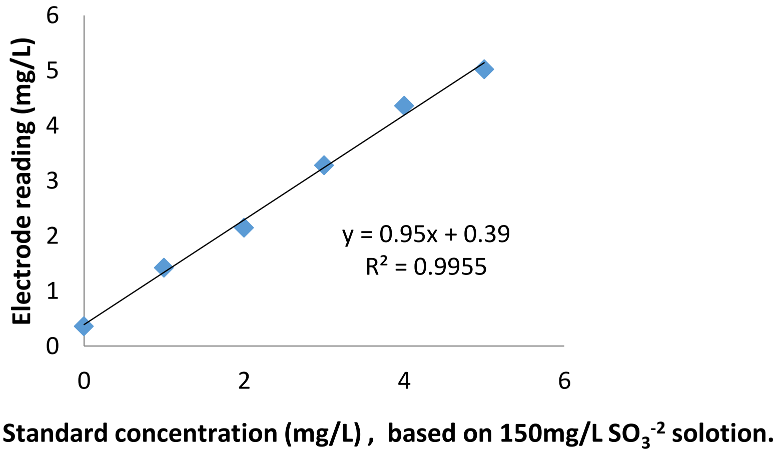

Supplementary Figure 8. Calibration curve of the YSI electrode against NH_3 standard solutions containing 150mg/L SO_3^{-2}.

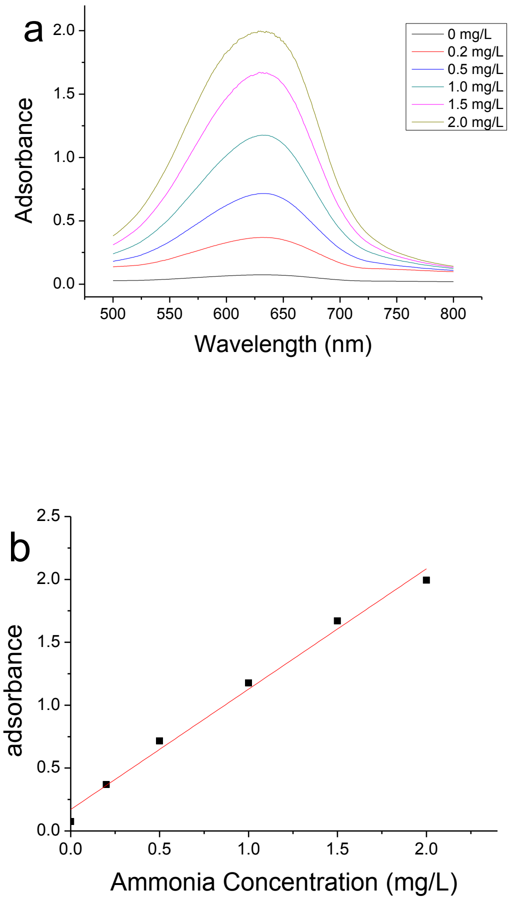

Supplementary Figure 9. (a) UV-Vis spectra for indophenol determination of standards ammonia. **(b)** Calibration curve obtained for standard ammonia solutions.

Supplementary Table1: Comparing ammonia yields in recent literature

year	paper	Absolute Yield (µmol)	Time (hour)	Type*	Reactor Vol. (ml)	Ref.
	This Work	1.88	24	I	10	
1994	Photocatalytic reduction of nitrogen over (Fe, Ru or Os)/TiO 2 catalysts	0.09	24	D	30	1
2013	Photo-illuminated diamond as a solid-state source of solvated electrons in water for nitrogen reduction	0.41	24	I	Not-mentioned	2
2013	Mechanism of N2 Reduction to NH3 by Aqueous Solvated Electrons	8.20×10^{-3}	24	D	10	3
2014	Plasmon - Induced Ammonia Synthesis through Nitrogen Photofixation with Visible Light Irradiation	6.00×10^{-3}	24	I	0.21	4
2015	Efficient visible light nitrogen fixation with BiOBr nanosheets of oxygen vacancies on the exposed {001} facets	5	1	D	100	5
2015	Photochemical Nitrogen Conversion to Ammonia in Ambient Conditions with FeMoS-Chalcogels	2.1	24	D	10	6

*(I): catalysts are immobilized on a substrate. (D): catalysts are dispersed and suspended in the solution. Units vary between journal articles, so, in order to minimize confusion, all the yields have been converted into µmol units. Other factors that effectively influence the yield calculation such as light intensity, yield physical shape (i.e., solid, gas), weight of semiconductor and physical shape of the catalyst, are all crucial when comparing the yield in different catalyst. The yield in this work is higher than the reported yields obtained on immobilized catalysts and highly competitive compared to the highest yields obtained by using dispersed photocatalysts.

Supplementary Table 2. Estimated maximum possible solar to NH_3 energy efficiencies according to reported data

To explore the possibility of coupling an electrolysis device with a separate PV cell, the following data from reports of recent electrochemical reduction reactions are combined with data for an appropriate PV cell that could drive the reaction. Since data for electrolysis inefficiencies are not available this data illustrates the maximum possible solar to ammonia energy efficiency in each case.

Ref (main text)	catalyst	media	T/^{o}C	P/ bar	Potential needed	N_2 Faradaic Eff/%	PV system*	PV efficiency % (max)	Maximum Solar to NH3 energy efficiency (%)
10	Polyaniline	Methanol/ $LiClO_4$/H+	25	1	-0.12V vs NHE (three electrodes)	1.3	4 single junction c-Si cell	10	0.13
10	Polyaniline	Methanol/ $LiClO_4$/H+	25	50	-0.12V vs NHE (three electrodes)	16.3	4 single junction c-Si cell	10	1.63
11	Pt/Nafion 211 Membrane	H_2O/air	RT	1	1.6 V (two electrodes)	1	4 single junction c-Si cell	10	0.1
12	Ni/Fe_2O_3	NaOH/KOH	200	25	1.2 (two electrodes)	35	3 single junction c-Si cell	13	4.55
*: ref to; Banerjee, A. *et al.* Photochemical Nitrogen Conversion to Ammonia in Ambient Conditions with FeMoS-Chalcogels. *Journal of the American Chemical Society* **137**, 2030-2034 (2015).									

Supplementary Note 1.

Alternative reactions utilising sulphite sources:

$$N_2 + 3H_2SO_3 + 3H_2O = 2H_2SO_4 + (NH_4)_2\,SO_4$$

$$N_2 + 3KHSO_3 + 3H_2O = KHSO_4 + K_2SO_4 + (NH_4)_2\,SO_4$$

Supplementary Note 2.

Calculation of the reaction free energy change (in the dark).

Using the overall reaction:

$3H_2O + 3SO_3^{2-} + N_2 + 2H^+ = 2NH_4^+ + 3SO_4^{2-}$ **Supplementary equation 1**

The free energy equation can be written

$\Delta G = \Delta G^0 + RT \ln [a(NH_4^+)^2 . a(SO_4{}^{2-})^3] / [a(H_2O). a(SO_3{}^{2-})^3. a(N_2) .a(H^+)^2]$

Supplementary equation 2

The following concentrations correspond to the situation in our experiments after 24hours reaction:

$[SO_3^{2-}]$ = 1.87mM

$[N_2]$ = 20mg/L = 0.71 mM (N_2 solubility in water)

$[H^+] = 1.5E^{-6}$ M

$[NH_4^+]$ = 0.18mM

$[SO_4^{2-}]$ = 0.27mM

We can calculate ΔG^0 for the reaction from the standard electrochemical potentials

$E^0(N_2/NH_4^+) = -0.09$ V

$E^0(H_2SO_3/H_2SO_4) = +0.17$ V

$=>\Delta E^0 = -0.26$

$\Delta G^0 = -nF\Delta E^0 = 150$ kJ/mol

where n = 6.

Substituting these values into Eqn 2 produces the result:

$\Delta G_{24\ hours}$ = 179 kJ/mol.

The positive value of ΔG indicates that the reaction is distinctly not spontaneous under these conditions in the dark.

Repeating the calculation for an earlier time where the concentration of products is only 10% of the t=24h values, the free energy change is then ΔG = 149 kJ/mol, confirming that the dark reaction is non-spontaneous even at quite early stages of the process.

Supplementary References:

1 Rao, N., Dube, S. & Natarajan, P. Photocatalytic reduction of nitrogen over (Fe, Ru or Os)/TiO_2 catalysts. *Appl. Catal., B* 5, 33-42 (1994).

2 Zhu, D., Zhang, L., Ruther, R. E. & Hamers, R. J. Photo-illuminated diamond as a solid-state source of solvated electrons in water for nitrogen reduction. *Nat Mater* 12, 836-841, (2013).

3 Christianson, J. R., Zhu, D., Hamers, R. J. & Schmidt, J. R. Mechanism of N_2 Reduction to NH_3 by Aqueous Solvated Electrons. *J. Phys. Chem. B* 118, 195-203 (2013).

4 Oshikiri, T., Ueno, K. & Misawa, H. Plasmon - Induced Ammonia Synthesis through Nitrogen Photofixation with Visible Light Irradiation. *Angew. Chem.* 126, 9960-9963 (2014).

5 Li, H., Shang, J., Ai, Z. & Zhang, L. Efficient Visible Light Nitrogen Fixation with BiOBr Nanosheets of Oxygen Vacancies on the Exposed {001} Facets. *Journal of the American Chemical Society* (2015).

6 Banerjee, A. *et al.* Photochemical Nitrogen Conversion to Ammonia in Ambient Conditions with FeMoS-Chalcogels. *J. Am. Chem. Soc.* 137, 2030-2034 (2015).
